# Biallelic Inactivating *TUB* Variants Cause Retinal Ciliopathy Impairing Biogenesis and the Structure of the Primary Cilium

**DOI:** 10.3390/ijms232314656

**Published:** 2022-11-24

**Authors:** Lucia Ziccardi, Marcello Niceta, Emilia Stellacci, Andrea Ciolfi, Massimo Tatti, Alessandro Bruselles, Cecilia Mancini, Lucilla Barbano, Serena Cecchetti, Eliana Costanzo, Marco Cappa, Mariacristina Parravano, Monica Varano, Marco Tartaglia, Viviana Cordeddu

**Affiliations:** 1 IRCCS-Fondazione Bietti, 00198 Rome, Italy; 2 Genetics and Rare Diseases Research Division, Ospedale Pediatrico Bambino Gesù, IRCCS, 00146 Rome, Italy; 3 Dipartimento di Oncologia e Medicina Molecolare, Istituto Superiore di Sanità, 00161 Rome, Italy; 4 Microscopy Unit, Core Facilities, Istituto Superiore di Sanità, 00161 Rome, Italy; 5 Unit of Endocrinology, Ospedale Pediatrico Bambino Gesù, IRCCS, 00165 Rome, Italy

**Keywords:** inherited retinal degeneration, whole exome sequencing, primary cilium, homozygous splice site variant

## Abstract

Inherited retinal degeneration (IRD) represents a clinically variable and genetically heterogeneous group of disorders characterized by photoreceptor dysfunction. These diseases typically present with progressive severe vision loss and variable onset, ranging from birth to adulthood. Genomic sequencing has allowed to identify novel IRD-related genes, most of which encode proteins contributing to photoreceptor-cilia biogenesis and/or function. Despite these insights, knowledge gaps hamper a molecular diagnosis in one-third of IRD cases. By exome sequencing in a cohort of molecularly unsolved individuals with IRD, we identified a homozygous splice site variant affecting the transcript processing of TUB, encoding the first member of the Tubby family of bipartite transcription factors, in a sporadic case with retinal dystrophy. A truncating homozygous variant in this gene had previously been reported in a single family with three subjects sharing retinal dystrophy and obesity. The clinical assessment of the present patient documented a slightly increased body mass index and no changes in metabolic markers of obesity, but confirmed the occurrence of retinal detachment. In vitro studies using patient-derived fibroblasts showed the accelerated degradation of the encoded protein and aberrant cilium morphology and biogenesis. These findings definitely link impaired TUB function to retinal dystrophy and provide new data on the clinical characterization of this ultra-rare retinal ciliopathy.

## 1. Introduction 

Inherited retinal degeneration (IRD) occurs in a clinically variable and genetically heterogeneous group of conditions characterized by photoreceptor degeneration and/or dysfunction [1]. The prevalence of monogenic IRD is estimated as 1 in 2000 individuals, affecting more than 2 million people worldwide [2]. This group of disorders typically present with severe vision loss that can be progressive, having an onset ranging from birth to late adulthood. Severe visual impairment affects mobility and independence, posing relevant psychological and economic burdens [3]. In the last ten years, genomic sequencing has allowed the identification of several genes implicated in IRD (for an updated list see the RetNet database, https://sph.uth.edu/retnet/). These genes encode proteins that have different roles in visual function, contributing to key processes in the cells of the pigmented cell layer and neurosensory retina [4,5]. Notably, retinal degeneration is frequently observed in ciliopathies, and a portion of IRD-related genes codifies for proteins involved in biogenesis and the maintenance of photoreceptor primary cilium [6,7,8,9]. Among these genes, *TUB* [MIM #601197], which encodes the first member of the Tubby family of bipartite transcription factors, has recently reported to be associated with a novel condition characterized by retinal dystrophy and obesity [MIM #616188] [10]. A truncating homozygous *TUB* variant [c.1194_1195delAG, p.Arg398Serfs*9] was identified in a single family with three affected members, suggesting loss of function (LoF) as the pathomechanism underlying the disorder. The TUB family of transcription factors counts four members in vertebrates (TUB, TULP1, TULP2 and TULP3), sharing a similar domain organization including a highly conserved C-terminal domain mediating DNA binding and a divergent N-terminal region encompassing the nuclear localization signal and the transcriptional activation domain implicated in protein–protein binding [11]. A conserved region at the N-terminus enables some members of the Tubby family, including TUB, to bind to the ciliary intraflagellar transport A protein (IFTAP) [12]. Similar to the other members of the TULP family, TUB is also implicated in the control of the initiation of phagocytosis, facilitating the removal of apoptotic cells or cellular debris by retinal pigmented epithelium (RPE) cells and macrophages [13]. Since the original report, no other subject carrying biallelic *TUB* variants has been described.

In this study, we report on an additional subject with retinal dystrophy carrying a homozygous LoF variant in *TUB*. In vitro analyses provide evidence of the disruptive functional impact of the variant on *TUB* transcript processing. We also show that the loss of TUB is associated with a defective cilium morphology and biogenesis in the primary patient’s fibroblasts. Our findings confirm the involvement of defective TUB function in retinal dystrophy and define this disorder as a novel ciliopathy. 

## 2. Detailed Case Description

**Figure 1:**
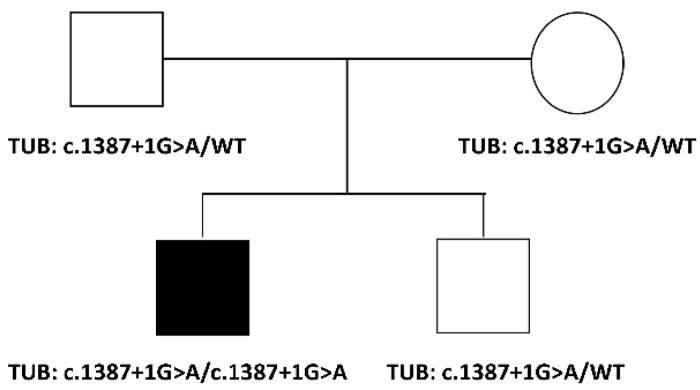
Family tree showing the segregation of the identified splice site change.

The proband is a 35-year-old male, the first child of healthy non-consanguineous parents of European descent (Figure 1). Family history was negative for retinal diseases or other congenital defects. The proband was born at term after an uneventful pregnancy. Length and weight at birth were reported as within the normal range. Apgar score was 8–10. No hearing impairment and/or learning difficulties were documented during the first year of age. 

At 5 years, the subject required consulting ophthalmologists for referring color blindness. A “blonde” aspect of the fundus oculi was observed and reduced full-field flash photopic and scotopic electroretinogram (ERG) responses were recorded. Visual acuity was not investigated at that time. Ophthalmological assessment at 26 years was suggestive of cone-rod dystrophy. Visual acuity was 0.8 Snellen in his right eye (RE) with myopic and astigmatic refractive error (RE: -4.50 dioptre sphere and -1 dioptre cylinder at 20°), and 0.2 Snellen in his left eye (LE) with myopic refractive error (LE: -2.00 dioptre sphere). Goldmann visual field was generally constricted, with reduced sensitivity in the superior central field in the RE and presented peripheral constriction and relative central scotoma in the LE. Light and dark adaptation sensitivity curves showed abnormal thresholds for both cones and rods. No number at the Ishihara charts were recognized. Intraocular pressure was within normal limits. The slit lamp examination biomicroscopy of the anterior segment was normal for cornea, iris and lens appearance. Fundus examination revealed bilateral optic disc pallor, depigmented aspect of the choroidal and retinal structures, diffuse retinal dystrophy with absence of intraretinal pigment dispersion or vitreous abnormalities and arteriolar attenuation. Full-field scotopic and photopic electroretinograms showed reduced a-wave and b-wave amplitude responses and delayed implicit times. Multifocal ERG ring analysis documented reduced response amplitude densities in all examined areas from the foveal center up to 20° of foveal eccentricity in both eyes (LE > RE). After reduced visual field in his LE, a large area of retinoschisis in the infero-temporal sector was observed by fundoscopy, which later progressed, resulting in full retinal detachment of the LE. The subject underwent to pars plana vitrectomy with silicon oil (30% polydimethylsiloxane, PDMS) tamponade. He underwent cataract surgery in his LE and to subsequent vitreo-retinal surgeries for relapsing retinal detachments in same eye (PDMS/perfluorocarbon liquid (PFCL)/PDMS tamponades exchange and intra-operatory laser treatment; PDMS/heavy silicon oil tamponades exchange and intra-operatory laser). One year later, he developed vitreo-retinal proliferation and retinal fibrosis in the LE, and visual acuity was hand motion; therefore, the subject underwent retinotomy and heavy silicon oil/PDMS tamponades exchange.

**Figure 2:**
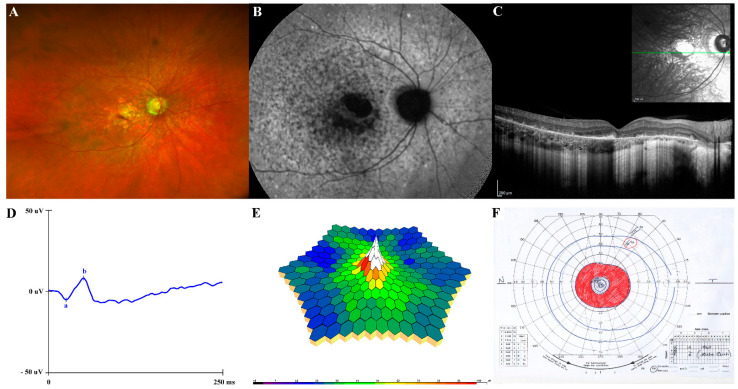
Ocular phenotype of the proband right eye. (**A**) Ultrawide field color fundus photograph. (**B**) The 55° fundus autofluorescence. (**C**) Spectral-domain optical coherence tomography line horizontal scan. (**D**) Full-field dark-adapted electroretinogram. (**E**) Three-dimensional multifocal electroretinogram plot. (**F**) Goldmann kinetic visual field.

At last evaluation (35 years), the subject presented with a good clinical condition. No cognitive deficit nor behavioral issues were observed, and no craniofacial dysmorphism and truncal obesity were noticed. An accurate endocrinological examination was uninformative. Anthropometric assessment displayed a height of 177.5 cm (+0.1 SD), weight of 95.70 kg (+1.6 SD), and OFC 60 cm (+0.2 SD) and BMI of 30.30 Kg/m^2^ (+1.6 SD). Tanner stage was Ph5Pg5, with a testicular volume 20 mL bilaterally. The subject’s visual acuity was 0.8 Snellen in his RE and light perception in his LE. Fundus oculi showed the unmodified condition of RE and diffuse chorio-retinal atrophy in the LE, as for visible vitreo-retinal surgical outcomes. Ultrawide field fundus photography showed macular atrophy in absence of peripheral pigment and confirmed a “blonde” aspect of the retina (Figure 2A). Fundus autofluorescence (FAF) revealed an hypoautoflorescent area in the macula due to RPE atrophy and hyper/hypoautofluorescence mottling in the posterior pole and immediately outside the vascular arcades (Figure 2B). The SD-OCT showed a disruption of the outer retina, including ELM, ellipsoid zone and RPE with outer retinal debris at the level of the RPE in the parafoveal region (Figure 2C). Full-field flash scotopic (Figure 2D) and photopic ERG confirmed reduced a-wave and b-wave amplitude responses and delayed implicit times in the RE. Similarly, multifocal ERG ring analysis, recordable only in the RE, showed the worsening of the response amplitude densities in all examined areas (0–20°) (Figure 2E), as compared to the previous examinations. Similarly, Goldmann visual field in his RE showed progressive constriction of the peripheral isopters and a ring scotoma enclosed between 10–20° of the visual field (Figure 2F); it was not executable, however, in his LE. Color blindness was also confirmed. Neck and abdominal ultrasound assessment did not reveal any abnormality. Measurements of basal hormones by the anterior pituitary, glycaemia and insulin were within the normal range. Basal plasma leptin level was in the low normal range, showing no leptin resistance. During his follow-up, clinical and ophthalmological assessments of other family members (parents and younger brother) were unremarkable.

## 3. Results

Trio-based WES allowed to identify a homozygous splice site *TUB* variant (chr11:8122545G > A, GRCh37.p13; c.1387 + 1G > A, NM_177972.3) as the only clinically and functionally relevant event putatively linked to the disorder (Supplemental Table S1). The variant had previously been annotated in public databases (dbSNP, rs1040410003) with low frequency (gnomAD v3.1, MAF = 0.00001972) at the heterozygous state. It was classified as likely pathogenic (PVS1/PM2/PP5) according to the American College of Medical Genetics and Genomics-Association for Molecular Pathology (ACMG-AMP) criteria (ClinVar: VCV000865971.1) [14]. Sanger sequencing confirmed the homozygosity for the variant in the proband and the heterozygous status in the healthy parents and unaffected sib (Figure 1). 

**Figure 3:**
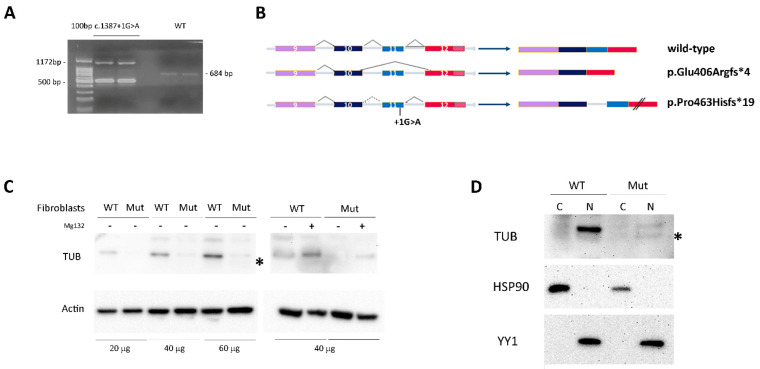
Characterization of the pathogenic *TUB* variant. (**A**) Transcript processing in primary skin fibroblasts from a healthy donor and from the proband carrying the homozygous splice site variant. Total RNA was extracted, reverse-transcribed and analyzed by PCR to resolve transcript processing. Two aberrant cDNA products were identified in mutated cells (left) compared to the expected length of the amplified fragment obtained from the control fibroblasts (right). (**B**) Schematic representation of aberrant transcript processing caused by the identified homozygous splice site change in *TUB*. (**C**) Western blot analysis of the TUB protein in patient’s fibroblasts and the control cells. Twenty, forty and sixty micrograms of whole cell extract were analyzed basally (left) and following 4 h treatment with 100 μM Mg132 (right) and probed with anti-TUB antibody (* indicates a non-specific product). (**D**) Expression of wild-type and mutant TUB protein was analyzed in the cytoplasmic and nuclear fractions were obtained from wild-type and mutated fibroblasts. Sixty micrograms of each fraction were analyzed by Western blot and then probed with anti-TUB antibody. Anti-YY1 and anti-HSP90 antibodies were used as the internal controls to assess the clarity of the different fractions (* indicates a non-specific product). Blots are representative of three experiments performed.

Since the variant was predicted to affect the exon 11 donor splice site, the total RNA from patient-derived skin primary fibroblasts was used to characterize the impact of the nucleotide change on transcript processing. The cDNA fragment encompassing the relevant exons was amplified, confirming the presence of two aberrantly spliced products (Figure 3A). The two aberrant PCR amplicons were isolated from agarose gel, purified and sequenced via Sanger. The former (500 bp) was derived from skipping of the penultimate exon (exon 11, ENST00000299506.3), resulting in a premature termination codon (i.e., p.Glu406Argfs*4), while the latter (1172 bp) was the result of retention of intron 10, resulting in a TUB protein having a divergent 19-residue-long C-terminus and lacking the last 44 amino acids (p.Pro463Hisfs*19) (Figure 3B). 

In the patient’s skin fibroblasts, the predicted TUB^Glu406Argfs*4^ protein was undetectable by Western blot [WB] analysis, while the TUB^Pro463Hisfs*19^ protein was poorly detectable likely due to accelerated degradation (Figure 3C, left panel). Consistently, the endogenous TUB^Pro463Hisfs*19^ level was partially rescued by blocking the proteasomal degradation pathway using MG132 (Figure 3C, right panel). 

**Figure 4:**
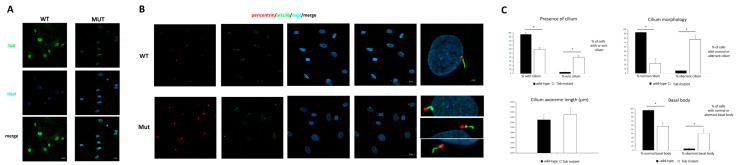
TUB subcellular localization and cilium defects in cells with TUB LoF. (**A**) Thirty-hour-starved and PFA-fixed fibroblasts from a healthy donor and from the proband were analyzed by confocal microscopy for the localization of TUB using antibodies against TUB. (**B**) The presence and morphology of primary cilia in starved fibroblasts from the proband and a healthy donor. Cells were starved for 30 h and fixed with PFA 4%. Primary cilia were analyzed using antibodies against ARL13B (cilium axonemal, green) and pericentrin (basal body, red) to investigate morphogenesis. Nuclei are DAPI stained (blue). Bars correspond to 20 and 2 μm (left panels). (**C**) Histograms represent the percent value of presence/absence of cilium, normal/aberrant cilium morphology and normal/aberrant basal body structure and the length (μm) of axoneme. Five hundred cells were counted for each condition and axoneme length was measured. Imagines and bar plots are representative of three experiments performed. * indicate statistical significance for *p* < 0.01 by using Student’s *T* test.

To investigate the cellular localization of the mutated protein, cytoplasm and nuclear cell fractions were obtained and analyzed by WB. The results demonstrate that both the wild-type (wt) and mutant TUB proteins were almost exclusively localized in the nucleus (Figure 3D). This finding was also confirmed by confocal microscopy analysis that evidenced the nuclear localization of the TUB mutant (Figure 4A).

Based on the documented role of TUB in early ciliogenesis [15], we assessed the localization of TUB at the cilium, but we failed to observe any consistent co-localization, possibly due to the low diffused levels of the protein in the cytoplasm To investigate the impact of defective TUB function on the morphogenesis of the primary cilium, a confocal analysis on primary fibroblasts was performed using antibodies against ARL13B, which localizes to primary cilia, and pericentrin, a component of the centrosome, which stains the basal body. As shown in Figure 4B, while almost all the control cells showed a primary cilium, 40% of the fibroblasts carrying the homozygous splice site variant in *TUB* did not show a primary cilium. Moreover, while no significant difference was observed in ciliary axoneme length between the starved control and mutant cells, a large percentage of the latter showed an abnormal cilium morphology and a poorly defined and disorganized basal body (Figure 4C).

## 4. Discussion

The loss of TUB function was recently reported to underlie a novel IRD condition based on the identification of homozygosity for a 2 bp deletion (c.1194_1195delAG) causing the premature termination of the protein (p.Arg398Serfs*9) shared by three affected members of a single consanguineous family [10]. While the combination of retinal dystrophy and early onset obesity can be reminiscent of other disorders (e.g., Bardet–Biedl syndrome (MIM#PS209900) and Alstrom syndrome (MIM#203800)), the absence of other clinically relevant feature/sign was suggestive of a previously unreported ciliopathy [10]. In this paper, we report on a second disruptive variant (c.1387 + 1G > A) affecting *TUB* in a subject with features that overlap with those previously described in the affected members of the original family. 

In the present and previously reported affected individuals, the ocular phenotype associated with defective TUB function is characterized by widespread retinal pigment epithelial (RPE) atrophy, deteriorating vision, the myopic and astigmatic refractive defect, the blonde fundus appearance, the ERG and OCT findings and progression of the disease (Figure 2). Notably, retinal detachment, without vitreous hemorrhage, was invariantly documented in the four individuals with biallelic inactivating *TUB* variants, suggesting that this feature likely represent a common finding in the disorder. 

Early onset obesity had previously been considered a major feature of the TUB-related condition [10], even though only two of three affected sibs were found obese (BMI > 30), and the glycemic and lipid profiles in one of affected sibs were found within the normal range. The finding of obesity in the apparently healthy father (heterozygous carrier) suggests a possible co-occurring genetic event contributing to obesity in the family. In the present report, the clinical assessment of the affected individual documented increased weight (95.7 kg, +1.6 SD) and BMI (30.3, +1.6 SD), thus indicating overweight, in absence of other signs of obesity. Although evidence suggests that a defective TUB function may result in disturbances in insulin and leptin activity and obesity in mice [16,17,18,19], based on the collected findings, the relevance of TUB in human obesity requires further study. To assess whether TUB LoF could impact on hypothalamic pathways, food intake and adiposity, the metabolic profile of the affected individual was investigated. Accurate endocrinological examination did not reveal any abnormality, and the measurements of basal hormones by the anterior pituitary, glycaemia, insulin and leptin were within the normal range, thus ruling out insulin or leptin resistance. Consistently, instrumental investigations (X-ray and ultrasound analyses) documented no sign of glycogen accumulation in the internal organs. Consistently, the absence of significant changes in lipid storage was demonstrated by thin-layer chromatography (HPTLC) analysis in proband-derived fibroblast (Figure S1), allowing to confirm an apparently normal endocrine–metabolic profile in the affected individual. Of note, the Tubby protein has been involved in cochlear degeneration [20,21]. Based on this functional link, we evaluated the occurrence of hearing problems and cochlear degeneration, which were not observed.

The homozygous *TUB* variant was predicted to affect transcript processing, causing the skipping of exon 11 and intron retention, resulting in a frameshift with the premature termination of the TUB protein. mRNA analysis in proband-derived fibroblasts confirmed a disruptive impact of the splice site change documenting two aberrantly processed transcripts predicted to encode a 406-residue-long protein (p.Glu406Argfs*4) that probably is not able to proceed in protein synthesis, and a 481-residue-long protein (p.Pro463Hisfs*19) characterized by a divergent C-terminus characterized by accelerated degradation (Figure 3B). These findings confirm LoF as the pathomechanism underlying this disorder.

Primary cilia are microtubule-based organelles that extend from the apical surface of the majority of mammalian cells. Cilia act as “cellular antennae”, receiving different inputs from the extracellular environment and transducing signals in response to stimuli [22]. A complex process required for proper cilia biogenesis function is the intraflagellar transport, which is required for assembling cilia and trafficking within primary cilia [23]. In this context, IFT-A has historically been believed to mediate retrograde intraflagellar transport inside the cilia and an IFT-A-binding domain at the TUB N-terminus has been demonstrated recently [24]. Moreover, co-localization between TUB and rootletin, the major structural component of the ciliary rootlet, has been observed in human retinal photoreceptors [10]. The use of a more informative in vitro model (e.g., induced pluripotent-stem-cell-derived retinal-pigment epithelium cells) and experimental strategies (e.g., siRNA-mediated TUB silencing) is required to appreciate more accurately the functional relevance of TUB in an appropriate cellular context. 

In summary, we confirm the link between TUB LoF and a new condition of human retinal ciliopathy and provide evidence of an altered cilium morphology/biogenesis in patient-derived fibroblasts. In fact, these cells displayed a reduced number of cilia when compared to the control cells, with a poorly defined and disorganized basal body structure, which are expected to prevent normal protein trafficking along the cilium. While these findings could explain the degeneration of photoreceptors characterizing this disorder, a relatively overall mild presentation has been associated with mutations in this gene. Multiple factors might contribute to this picture. First, the functional relevance of TUB in the context of ciliogenesis and cilium function might vary in different cell lineages. Second, functional compensation exerted by other related proteins might contribute to buffer the severity of the endophenotype. Third, specific cell lineages (e.g., rod and cone cells) might be particularly sensitive to TUB LoF. The collection of additional patients with biallelic variants in the gene will allow a more accurate characterization of the clinical spectrum associated with TUB LoF. Based on these considerations, TUB loss of function could be considered as responsible for a novel form of isolated retinal ciliopathy.

## 5. Conclusions

In this paper, we confirm that biallelic inactivating variants in TUB cause a retinal disorder and functionally associate this condition with the occurrence of defects of the basal body structure and axoneme morphology of the primary cilium and overall defective ciliogenesis. Although the present findings suggest that TUB plays a role in the pathogenesis of retinal ciliopathies, dedicated functional studies are required to profile the mechanism of disease. This work confirms the clinical relevance of biallelic LoF variants of *TUB*, which should be considered in clinical settings and the genetic diagnosis of IRD disorders. 

## 6. Methods

The present research followed the tenets of the Helsinki Declaration (1964 and further revisions) and the study was approved by the local ethics committee (Comitato Etico Centrale IRCCS Lazio, Sezione IFO/Fondazione Bietti, Rome, Italy). Upon recruitment in the study (FB RET-02-2019), executed from February to July 2019 at the IRCCS Fondazione Bietti, informed consent after full explanation was obtained from each subject included in the study.

## 7. Ophthalmological Evaluation

The proband and his family underwent comprehensive ophthalmological examination including visual acuity measured by the Early Treatment Diabetic Retinopathy Study (ETDRS) charts (Lighthouse, Low Vision Products, Long Island City, NY, USA) at the distance of 4 m and expressed as the logarithm of the minimum angle of resolution (logMAR); intraocular pressure (IOP) measurement; anterior segment observation by slit-lamp biomicroscopy and fundus examination by indirect ophthalmoscopic examination using + 90D non-contact lens (Volk Optical, Mentor, OH) after pupillary dilatation with tropicamide 1% drops. Goldmann visual field was assessed by kinetic Goldmann perimeter Haag-Streit 940. Chromatic test was evaluated by the monocular administration of Ishihara pseudoisochromatic plates (24 plates edition, Kanehara Trading Inc., Tokyo, Japan) in natural daylight. Electrophysiological assessment included dark-adapted and light-adapted full-field electroretinogram (Retimax Advanced Plus apparatus, CSO, Firenze, Italy) and multifocal electroretinogram (mfERG; VERIS Clinic TM version 4.9; Electro-Diagnostic Imaging, San Mateo, CA, USA) recordings according to the 2011 International Society for Clinical Electrophysiology of Vision (ISCEV) [25] by using the Dawson–Trick–Litzkow (DTL) contact electrodes and having the pupil dilated at 8 mm.

Retinal imaging was executed by ultrawide field fundus photograph obtained with Optos California (Optos PLC, Dunfermline, Scotland, United Kingdom); FAF was also obtained during the same examination (488 nm excitation, barrier filter transmitted light from 500 to 680 nm, 55°) using Heidelberg Spectralis OCT (HRA + OCT, Heidelberg Engineering, Heidelberg, Germany). SD-OCT scan pattern was acquired with a minimum of a 20 × 20 degree rectangle centered on the fovea and 25-line B-scans spaced 235 μm apart and composed of 50 averaged frames by using the same instrument.

## 8. Molecular Analyses

Karyotype, CGH array and mutation scan by parallel sequencing considering a retinal cone–rod dystrophy panel of 70 genes were informative. Genomic DNA was collected after obtaining written informed consent. Whole-Exome sequencing (WES) and data analysis were performed as previously reported [26,27] (Supplementary Table S1). DNA of the affected subject and his parents was extracted from circulating leukocytes and sequenced using Illumina paired-end technology by means of SureSelect Human All Exon v.7 (Agilent, Santa Clara, CA, USA) kits. WES raw data were processed and analyzed using an in-house implemented pipeline as previously described [28,29], according to the GATK’s Best Practices [30]. The UCSC GRCh37/hg19 version of genome assembly was used as a reference for read alignment by means of BWA-MEM [31] tool and the subsequent variant calling with HaplotypeCaller (GATK v3.7) [28]. Variants’ functional annotation was made with the SnpEff v.5.0 [32] and dbNSFP v.4.2 [33] tools. Relevant in silico impact prediction tools, such as Combined Annotation Dependent Depletion (CADD) v.1.6 [34], Mendelian Clinically Applicable Pathogenicity (M-CAP) v.1.0 [35] and InterVar v.2.0.1 [36], were also used. By filtering against our population-matched database (~2500 WES) and public databases (dbSNP150 and gnomAD v.2.0.1), the analysis focused on high-quality rare variants that affect coding sequences and splice site regions. Sanger sequencing was performed for variant validation and segregation analysis (primers available upon request). 

## 9. Functional Studies

### 9.1. RT-PCR Analysis

Proband skin biopsy was obtained after informant consent was secured. Total RNA was extracted from the proband and healthy donor fibroblasts by using RNeasy Mini Kit (Qiagen, Hilden, Germany), according to the manufacturer’s recommendations. Total RNA concentration and quality were assessed by measuring the absorbance at 260 and 280 nm. Reverse transcription was carried out using a SuperScript RT reagent Kit (Invitrogen; ThermoFisher Scientific, Waltham, MA, USA). The obtained cDNA pools were amplified by PCR using specific primers located in exons 10 and 12 of the *TUB* gene, and PCR products were separated on a 2% agarose gel. The sequences of the primers are available upon request.

### 9.2. Cell Cultures and In Vitro Studies

Primary skin fibroblasts were cultured in DMEM supplemented with 10% heat-inactivated FBS, 2 mM glutamine, 100 units/mL of penicillin and 100 µg/mL of streptomycin, and maintained at 37 °C in a humidified atmosphere containing 5% CO_2_. After treatment, fibroblasts were lysed in RIPA buffer supplemented with phosphatase and protease inhibitors. Lysates were kept on ice (30 min) and then centrifuged at 16,000× *g* (20 min, 4 °C). Supernatants were collected and protein concentration was determined by Quick Start Bradford Dye Reagent (Bio-Rad Laboratories, Hercules, CA, USA), using bovine serum albumin (BSA) as a standard. Fibroblast homogenates were resolved by SDS PAGE and transferred to nitrocellulose membranes (Bio-Rad Laboratories). Blots were blocked for 1 h with 5% non-fat milk powder in phosphate-buffered saline (PBS) containing 0.1% Tween-20 and incubated overnight with specific antibodies. Anti-TUB primary polyclonal antibody (1:1000, Invitrogen; ThermoFisher Scientific) and goat anti-rabbit-HRP IgG secondary antibody (Invitrogen; ThermoFisher Scientific) were diluted in blocking solution. Immunoreactive proteins were detected by an ECL SuperSignal West Femto Maximum Sensitivity Chemiluminescent Substrate, according to the manufacturer’s instructions (Thermo Scientific, USA).

### 9.3. Confocal Laser Scanning Microscopy

Approximately 3 × 10^4^/mL fibroblasts were seeded on glass coverslips and maintained in culture in complete medium for 24 h. Subconfluent fibroblasts were starved for 30 h and then fixed with 4% paraformaldehyde. Subsequently, cells were stained with rabbit anti-TUB (1:100 dilution), rabbit polyclonal anti-ARL13B (1:100, Abcam, Cambridge, UK) and mouse monoclonal anti-Pericentrin (1:100, Abcam) antibodies followed by goat anti-rabbit Alexa Fluor 488 and goat anti-mouse Alex Fluor 594, respectively (1:200 dilution; Molecular Probes, Eugene, OR, USA). After staining, coverslips were extensively rinsed and mounted onto microscope slides using Vectashield with DAPI mounting medium (Vector Laboratories, Newark, CA, USA). Analyses were performed in three independent experiments on a Zeiss LSM980 (Zeiss, Oberkochen, Germany) using a 63×/1.4 N.A. oil objective and excitation spectral laser lines at 405, 488 and 594 nm. Five hundred cells were counted for each condition in each experiment, and axoneme length was measured for each cell by using Zen Blue 3.2 software. Image acquisition and processing were performed as previously reported [37].

### 9.4. Lipid Extraction and Analysis

Control and mutated fibroblasts cultured in 150 cm^2^ dishes were harvested using trypsin after reaching confluence, rinsed with phosphate-buffered saline (PBS) and pelleted by centrifugation. Cell pellets were resuspended in 1 mL 0.9% NaCl and 6 mL of chloroform/methanol (2:1, v/v) were added. Mixtures were vortexed vigorously and centrifuged at 1500× *g* for 15 min; the aqueous phase was discarded and organic phase was dried under N_2_ gas. Lipid extracts from 1.5 × 10^6^ cells were applied on high-performance thin-layer chromatography (HPTLC) silica gel 60 plates (Merck, Darmstadt, Germany). Neutral lipids were resolved using a solvent system of hexane/diethyl ether/acetic acid (70:30:1, v/v/v) and were detected by staining with an aqueous solution containing 3% cupric acetate and 8% phosphoric acid and subsequent charring at 140 °C for 10 min. Lipids were identified by the co-migration of commercially available standards.

URLs.*Online Mendelian Inheritance in Man [OMIM], http://www.ncbi.nlm.nih.gov/omim/;

NCBI Gene, http://www.ncbi.nlm.nih.gov/gene/;

Picard tools, http://picard.sourceforge.net/;

wAnnovar, http://wannovar.usc.edu/;

dbSNP137, http://www.ncbi.nlm.nih.gov/projects/SNP/snp_summary.cgi/;

1000 Genomes Project, http://www.1000genomes.org/;

HapMap Project, http://hapmap.ncbi.nlm.nih.gov/;

SMART, http://smart.embl-heidelberg.de/;

Protein Data Bank [PDB], http://www.rcsb.org/pdb/home/home.do.

## Data Availability

Data that support the findings of this study are available from the corresponding authors upon request.

## Supplementary Materials

The following supporting information can be downloaded at: https://www.mdpi.com/article/10.3390/ijms232314656/s1.
