# Supplementary material for: Biallelic Inactivating TUB Variants Cause Retinal Ciliopathy Impairing Biogenesis and the Structure of the Primary Cilium
Source: Int J Mol Sci. 2022 Nov 24;23(23):14656. doi: 10.3390/ijms232314656 (PMC9740599; doi:10.3390/ijms232314656)
Supplement: Supplementary file 1 [file ijms-23-14656-s001.zip › Suppl materials/suppl material.zip!/suppl material/Supplem material.pdf]

Supplemental data

Table S1. WES data output.

	Proband
WES enrichment kit	Agilent SureSelect Human All Exon V7
Total passing quality filters unique reads	144,793,449
Target regions coverage >10x	91.2%
Target regions coverage >20x	90.0%
Average sequencing depth on target	157x
Private and low frequency variants with functional effect[1]	252
Putative disease genes [AD trait][2]	5[3]
- candidate genes	0
Putative disease genes [AR/X-linked trait][2]	4[4]
- candidate genes	1, *TUB*

[1]Private or low frequency [gnomAD MAF<0.1% and recurrence <1% within our ~2,500 exomes database] non-synonymous single nucleotide variants or indels within coding exons and splice regions [-3/+8].
[2]Functional impact assessed by Combined Annotation Dependent Depletion [CADD] v.1.6 [http://cadd.gs.washington.edu/], Mendelian Clinically Applicable Pathogenicity [M-CAP] v.1.0 [http://bejerano.stanford.edu/mcap/] and InterVar [http://wintervar.wglab.org] v2.0.1. Variants predicted as benign or likely benign by InterVar were discarded and only those with CADD score>20 or M-CAP score>0.025 were retained.
3BBS4 [c.857T>C; p.Met286Thr], VPS13B [c.4786C>T; p.Leu1596Phe], IRX5 [c.760G>A; p.Glu254Lys], DRD4 [c.719G>C; p.Gly240Ala], LAMA1 [c.1745C>T; p.Ala582Val].

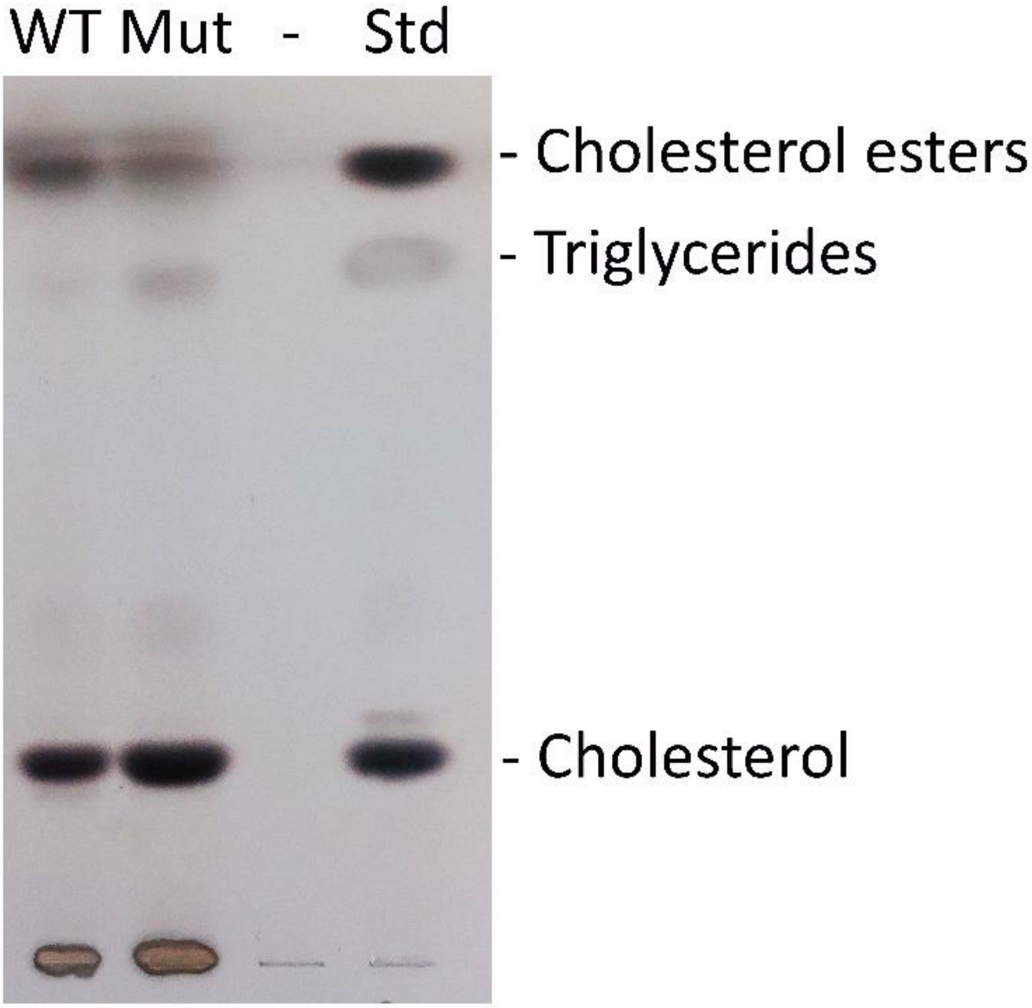

<u>Figure S1</u>. Lipid analysis in control and patient fibroblasts.

Analysis of neutral lipids were performed by high-performance thin-layer chromatography [HPTLC] using the appropriate solvent mixture. As standards [Std], free cholesterol, cholesterol ester, triglycerides were utilized. The position of pure standard lipids is indicated on the margin of the panel.
